# Association of Cost and Medical Service Satisfaction with Korean and Conventional Medicine Use before and after Surgery in Postsurgical Patients: A Questionnaire Survey of Korean Patients with Postsurgical Pain Visiting Korean Medicine Hospitals

**DOI:** 10.1155/2020/8195241

**Published:** 2020-03-19

**Authors:** Ki-Beom Lee, Yoon Jae Lee, Me-riong Kim, Kyung-Min Shin, In-Hyuk Ha

**Affiliations:** ^1^Jaseng Hospital of Korean Medicine, Seoul, Republic of Korea; ^2^Jaseng Spine and Joint Research Institute, Jaseng Medical Foundation, Seoul, Republic of Korea; ^3^Clinical Medicine Division, Korea Institute of Oriental Medicine, Daejeon, Republic of Korea

## Abstract

This study aimed to assess the costs, health status, and medical service satisfaction with Korean and conventional medicine use before and after surgery of patients visiting Korean medicine hospitals for postsurgical musculoskeletal pain. The study population comprised patients who visited KM hospitals for the first time between June and November 2017 for persistent or recurrent pain and discomfort after low back, neck, shoulder, or knee surgery. Various validated questionnaires were used to collect data. A total of 100 participants were enrolled, and the majority had undergone low back surgery (*n* = 82). The participants had received 1.3 ± 0.7 magnetic resonance imaging (MRI) examinations and 2.4 ± 2.8 X-rays before surgery. Conventional interventions used before surgery were physical therapy (43%), medications (34%), and injections (28%), in descending order, while 48% of patients reported having received acupuncture 51.3 ± 81.1 times. The mean satisfaction score for surgery was 5.5 ± 2.8 points based on a 9-point Likert scale, while that for KM-based interventions was 6.3 ± 1.7 points. With respect to health-related information, the mean scores were 6.0 ± 2.2 points on the Numeric Rating Scale (NRS), 0.6 ± 0.2 points on the 5-Level EuroQol-5 Dimension (EQ-5D-5L), and 15.3 ± 10.2 on Beck's Depression Index II (BDI-II). The mean score on the Oswestry Disability Index (ODI) in patients with low back pain was 40.1 ± 19.2 points. Work impairment, as measured using the Work Productivity and Activity Impairment Questionnaire: General Health (WPAI-GH), was 62.5 ± 47.8%, while activity impairment was 5.9 ± 2.6%. Participants tended to show low satisfaction regarding surgery and high preference for KM-based interventions. In particular, low back surgery patients reported high ODI scores, indicating high dysfunctional levels and poor prognosis after surgery. It can be inferred that it is therefore important to provide appropriate presurgical and postsurgical care for patients with musculoskeletal pain to improve pain, function, and quality of life.

## 1. Introduction

Musculoskeletal disorders refer to conditions in which pain or dysfunction in the muscles, ligaments, bones, or joints has occurred, generally due to injury or accumulative trauma, and low back and neck pain are reported to be the leading causes of disability and decreased quality of life [1]. Currently, chronic pain treatments include medications, such as opioids, nonsteroidal anti-inflammatory drugs (NSAIDs), antidepressants, anticonvulsants, and muscle relaxants, and nonpharmacological interventions, such as injections, implantable devices, and surgery. Various treatment modalities may be used in conjunction for more effective pain reduction [2].

Patients who are unable to achieve satisfactory pain relief using nonsurgical interventions often turn to surgical means. This behavior is partly reflected in the increasing trends in orthopedic surgery in Korea, where the number of spinal and joint surgery cases is steadily increasing. There was a 1.4-fold increase in the number of general spinal surgeries between 2007 and 2015; a 4-fold increase in the number of knee arthroplasties between 2001 and 2010; and a 10-fold increase in the number of rotator cuff surgeries between 2007 and 2015, respectively [3, 4].

While prevalence of spine and joint surgery steadily increases, many patients continue to suffer from persistent or recurrent pain after surgery, which incurs considerable medical expenditure and social burden. When such pain persists for 3 months or longer, it is referred to as chronic postsurgical pain (CPSP) [5, 6]. While the etiology and risk factors of CPSP have not yet been clearly identified, approximately 10–50% of patients undergoing surgery are known to experience CPSP [7]. Of surgery types, risk of CPSP is roughly 3 times higher for orthopedic surgery than other surgery types [8], which acts as an added difficultly in achieving satisfactory relief from pain and discomfort after orthopedic surgery.

CPSP can lead to decreased satisfaction regarding medical services due to impeded postsurgical rehabilitation and decreased quality of life [9]. Medical service satisfaction is influenced by expectations patients have about received medical services, and postsurgical pain can be a major cause of dissatisfaction, especially in orthopedic surgery, as the main expectation from surgery in orthopedic surgery cases would be pain relief [10].

In Korea, the cost of surgery for disc disorders or stenosis is more than 9 times that of nonsurgical interventions [11], and CPSP increments the burden of medical expenses. In a US questionnaire survey on patients with posttraumatic and postsurgical neuropathic pain, the financial burden was reported to increase in linear fashion with pain intensity [12].

In many countries, including Korea, Japan, and China, complementary and alternative medicine (CAM) is used to treat pain, including postsurgical pain [13]. Conventional medicine and traditional Korean medicine (KM) coexist within the dual medical system in Korea, and, as a type of CAM, KM covers such treatments as acupuncture, cupping, moxibustion, pharmacopuncture, Chuna manual medicine, and herbal medicine. Patient satisfaction with CAM is generally high [14], with some patients seeking CAM due to dissatisfaction with conventional medicine treatment and outcomes [3]. In a questionnaire-based study on CAM (KM) in Korea, 47% of the respondents reported that they were equally satisfied with conventional medicine and CAM (KM), while 25% reported higher satisfaction with CAM (KM) [15].

Studies on medical service satisfaction and costs in CPSP patients are few, and those on usage and preference regarding integrative medicine or conventional and nonconventional medicine use are even scarcer. The present survey study therefore employed various validated questionnaires to comprehensively assess medical expenditure and satisfaction with regard to conventional and Korean medicine use in postsurgical patients who chose to visit KM hospitals for postsurgical musculoskeletal pain in Korea.

## 2. Materials and Methods

### 2.1. Characteristics of Participants and Research Institution

The present study was designed as a multicenter, cross-sectional survey study targeting patients visiting KM hospitals for postsurgical musculoskeletal pain treatment. Jaseng Hospital of Korean Medicine (JHKM) is a spine-specialty hospital that employs integrative medicine consisting of conventional and Korean medicine [16], and the present study recruited patients from the main branch of JHKM located in Seoul and three branches located in Daejeon, Bucheon, and Busan, respectively. Inclusion criteria included patients with history of low back, neck, knee, or shoulder surgery visiting KM hospitals for the first time between June and November 2017. No promotional or advertising campaigns were conducted to recruit participants, and most participants visited JHKM based on personal choice and preference.

### 2.2. Procedures and Methods for Data Collection

The preliminary consultation process was similar for all patients. As such, all patients with a chief complaint of low back, neck, knee, or shoulder pain with history of surgery were considered to be eligible. Among these patients, those who voluntarily consented to participate in the study were interviewed by the clinical investigator from each center to determine whether they met the inclusion/exclusion criteria. The inclusion criteria were as follows: having a chief complaint(s) of at least one or more of the following: low back pain (or radiating leg pain), neck pain (or radiating arm pain), knee pain, or shoulder pain; having a history of musculoskeletal surgery related to the chief complaint(s); being capable of effectively communicating with the researcher(s) and understanding the survey items; and providing written informed consent. The exclusion criteria were as follows: having no history of musculoskeletal surgery associated with the low back, neck, shoulder, or knee region(s); having pain that was mainly attributed to a traffic accident injury; being incapable of answering the interview and survey items; and having any other reason rendering study participation inappropriate, as judged by the researcher(s).

### 2.3. List of Data Collected

Participants completed a questionnaire containing items about demographic information, surgery-related information, medical cost, and health status. Each participant required approximately 30 minutes to complete the questionnaire, and data for each item were collected by researchers following preliminary consultation, screening, and written informed consent and prior to treatment according to a predetermined standard operating procedure.

### 2.4. Sociodemographic and Surgery-Related Information

For identification of basic information about the participants, data regarding age, sex, height, and weight were collected. Comorbidities consisted of only diseases that were being treated or controlled by medication at the time of the survey, including hypertension, diabetes, depression, cardiovascular disease, pulmonary disease, and gastrointestinal disease. Data were also collected on occupational status, alcohol consumption status, standardized alcohol consumption amount according to standards set by the Korea Ministry of Food and Drug Safety, smoking status, and amount of smoking. The site of previous surgery and site of major pain at the time of survey were checked. The participants were also asked to provide responses on the cause of pain that led to the initial surgery and associated medical costs.

### 2.5. Medical Costs before and after Surgery

For in-depth analysis of the details of utilization of medical resources at different periods, the participants were instructed to provide answers to each question on utilization of medical resources before and after the initial surgery. The items were standardized for all pain regions, and participants were instructed to provide answers to questions pertaining to diagnoses, tests, and treatments involving the initial surgery site. Items were divided into conventional diagnostic tests, conventional interventions, KM diagnostic tests, and KM interventions. A list identifying Korean national health insurance coverage and noncoverage items was created. For participants who underwent two or more surgeries, “resurgery” was selected from the list of conventional interventions after surgery. For each medical service item, data were collected on the number of times the service was used and the mean cost per instance of service use. Data for conventional medications and herbal decoctions were calculated as daily cost.

### 2.6. Outcome Measures: Medical Service Satisfaction and Health Information

To measure the medical service satisfaction of the participants with respect to the following five categories: initial surgery, conventional diagnostic tests, conventional interventions, KM diagnostic tests, and KM interventions, questions based on a 9-point Likert scale were used. The question asked was, “How helpful do you think this test or treatment was to your health? Please respond with 1 point for not helpful at all to 9 points for very helpful.” The participants who did not receive that particular test or treatment were also allowed to answer the question, to help determine individual preferences.

To assess the current health status, quality of life, and degree of dysfunction of the participants, a health-related questionnaire survey was conducted. The survey used the Numeric Rating Scale (NRS), 5-Level EuroQol-5 Dimension (EQ-5D-5L), Beck's Depression Index II (BDI-II), and Work Productivity and Activity Impairment Questionnaire: General Health (WPAI-GH) scale. The NRS is widely used to assess the level of pain in patients with chronic pain [17]. It employs an 11-point scale for rating the “level of pain felt while performing daily activities today.” The EQ-5D-5L [18] comprises descriptive 5-level scale items and the EuroQol-Visual Analogue Scale (EQ-VAS). The present study used the validated Korean version of the EQ-5D-5L questionnaire provided by the EuroQol Group and the descriptive scale with formulas validated for the Korean population in calculating total scores.

For functional assessment of the low back, neck, knee, and shoulder, validated Korean versions of the Oswestry Disability Index (ODI) [19], Neck Disability Index (NDI) [20], Western Ontario and McMaster Universities Arthritis Index (WOMAC) [21], and Shoulder Pain and Disability Index (SPADI) [22] were used, respectively. For these assessments, participants responded only to the scale that corresponded to their respective surgery site. A validated Korean version of the BDI-II [23], translated by Korean researchers, was used for the clinical assessment of depression. In keeping with recommendations of the Institutional Review Board (IRB) at JHKM, participants who were found to show borderline clinical depression or higher were told that they may need psychiatric counseling, separately from the study. The WPAI-GH is designed to estimate the influence of health status and disease-related symptoms on daily activity and work performance [24]. The present study used the officially supplied 6-item Korean version. The survey results were converted into the daily activity impairment percentage index calculated based on all patients' scores and the work impairment percentage index calculated from participants who were actively employed.

### 2.7. Data Analysis

The study was initially planned to classify participants into 4 groups according to surgery site (low back, neck, knee, and shoulder). However, as over 80% of participants was low back surgery patients, the patient population was dichotomized into the low back surgery and other surgery patient groups in actual analysis. The data from the completed questionnaires were processed by double entry method by two researchers (JHK, HYM). IBM SPSS version 25 was used to conduct the statistical analysis (SPSS Inc., Chicago, IL). Categorical variables were expressed as frequency and percentage, while continuous variables were expressed as mean ± standard deviation. *P* < 0.05 was considered to be statistically significant.

## 3. Results

**Figure 1:**
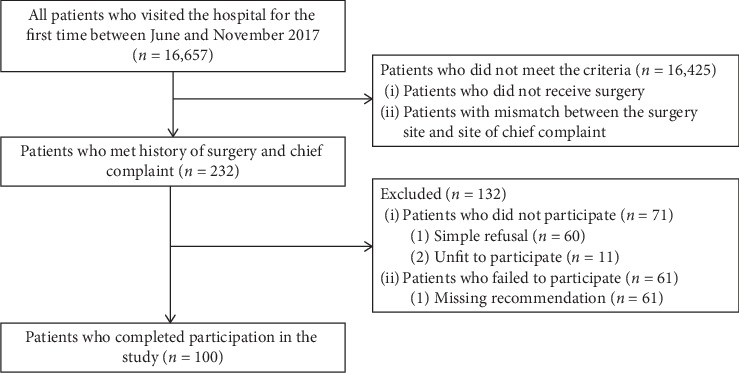
Flowchart of the study.

A total of 16,657 patients visited one or more of the four KM hospital study sites during the study period. Among these patients, 232 were identified as having a past history of surgery that matched the site of their chief complaint. After excluding patients who did not consent to study participation or those who could not be included due to noncompliant research schedules, 100 patients (low back: 82l; neck: 7; knee: 10; and shoulder: 1, respectively) participated in the study and completed the questionnaires (Figure 1).

The sociodemographic characteristics are presented in Table 1. The mean age was 53.2 ± 15.8 years in the low back surgery group and 49.8 ± 13.6 years in the other surgery (including neck, shoulder, and knee surgery) groups. Most patients in the low back surgery group were in the 60–69 year age range, while most in the other surgery groups were in their 50s. Males accounted for 52% of the study population, and the morbidities of highest frequency were hypertension (31%), diabetes (11%), and cardiovascular disease (11%). Roughly half of the participants were currently employed. The main cause of pain episode onset for each surgery group was daily activity (59.8% and 55.6%, respectively), work or exercise (31.7% and 27.8%, respectively), and accidents (8.5% and 16.7%, respectively). The cost of initial surgery was 4,217.3 ± 2,770.4 and 3,635.0 ± 2,052.2 thousand Korean Won in the low back and other surgery groups, respectively, indicating that costs for low back surgery were higher.

The results on utilization of medical resources and cost burden were divided into “before” and “after” surgery, which are presented in Tables 2 and 3, respectively.

### 3.1. Utilization of Medical Resources and Cost Burden before Surgery

Among conventional tests before surgery, magnetic resonance imaging (MRI) examination was used for diagnosis in most patients (89%), while X-ray and CT examinations were used on 60% and 36% of the patients, respectively. The mean number of MRI and X-ray examinations performed per person was 1.3 ± 0.7 and 2.4 ± 2.8 times, respectively, while the cost burden per MRI examination was 547.9 ± 317.9 thousand Korean Won per person. The most common conventional intervention used before surgery was physical therapy (43%), followed by medications (34%) and injections (28%), respectively. Utilization of minimally invasive surgical treatments was 11.0% and 11.1% in the low back and other surgery groups, respectively, showing a very small difference between the two groups. Meanwhile, the cost of minimally invasive surgical treatments was 3,500.0 ± 2,107.1 and 1,325.0 ± 671.8 thousand Korean Won in the low back and other surgery groups, respectively, showing that the cost burden was relatively high in the low back surgery group. The most common KM intervention used before surgery was acupuncture (48%), and users reported acupuncture use of 51.3 ± 81.1 times. Other KM intervention use was in the following order: cupping (35%), pharmacopuncture (17%), and herbal decoction (14%). Herbal decoction had the highest cost burden of KM interventions: approximately 3,229.4 ± 4,248.0 thousand Korean Won over a course of 98.9 ± 106.7 days per person.

### 3.2. Utilization of Medical Resources and Cost Burden after Surgery

Utilization of medical resources and cost burden after surgery were based on medical services received by the patients from the end of the initial surgery to the day of the survey. Among conventional tests after surgery, MRI (68%) and X-ray (52%) examinations were used most often. The most common conventional intervention used after surgery was medication (57%), with participants reporting intake over 126.4 ± 351.7 days. Other high frequency interventions included physical therapy (46%) and injections (39%), respectively. Meanwhile, although only 25% of the participants had used conventional manual therapy, the cost burden was high at 1,784.6 ± 4,040.2 thousand Korean Won per person. Furthermore, only 6% of the participants responded that they had received minimally invasive surgical treatments after the initial surgery (mean: 2.8 times), while 23% of the participants had experienced resurgery (mean: 1.3 times). The mean cost burden associated with such minimally invasive surgical treatments and resurgery was 5,783.3 ± 2,958.7 and 4,543.2 ± 4,021.9 thousand Korean Won, respectively. With respect to KM interventions after surgery, 52% of the participants responded that they received 114.2 ± 422.3 acupuncture treatments, with a mean cost burden of 627.0 ± 2,576.4 thousand Korean Won. The KM intervention with the highest cost burden per person was herbal decoction, with 18% of the participants using this intervention at a cost burden of 1,880.0 ± 2,180.1 thousand Korean Won per person.

The study also investigated patient preferences for medical service items based on their background knowledge or experience in KM and/or conventional medicine use, including surgery (Table 4). The mean patient satisfaction score for surgical outcomes was 5.5 ± 2.8 points based on a 9-point scale. The mean satisfaction score for conventional diagnosis, conventional interventions, KM diagnosis, and KM interventions was 6.4 ± 2.4, 5.2 ± 2.0, 5.5 ± 1.8, and 6.3 ± 1.7 points, respectively. Moreover, the mean NRS score, indicating the level of pain reported by participants, was 6.0 ± 2.2 points, while the mean EQ-5D-5L and EQ-VAS score was 0.6 ± 0.2 and 53.0 ± 20.3 points, respectively. Meanwhile, the mean BDI-II score was 15.3 ± 10.2, 15.1 ± 10.5, and 16.2 ± 9.3 points across the whole study population, the low back surgery group, and other surgery group, respectively. The dysfunction scores were measured separately for each surgery site, and the results showed a mean ODI score of 40.1 ± 19.2 for low back, mean NDI score (*N* = 7) of 33.1 ± 19.9 points for the neck, mean WOMAC score (*N* = 10) of 37.8 ± 20.3 points for the knee, and mean SPADI score (*N* = 1) of 81.5 points for the shoulder. All items on the WPAI-GH were answered by the participants who were employed, and the resulting work impairment score was 62.5 ± 47.8%. The activity impairment score for all participants was 5.9 ± 2.6%.

## 4. Discussion

The objective of the present investigator-initiated, multicenter study was to assess the perisurgical medical expenditure, health status, and medical service satisfaction in patients with postsurgical musculoskeletal pain visiting KM hospitals in Korea after orthopedic surgery. JHKM, where the study was conducted, is the largest KM institution in Korea and treats more than 900,000 spinal disorder cases annually as a spine-specialty hospital designated as such by the Korean Ministry of Health and Welfare [16], and the study population was set as patients with chief complaint of pain at the surgery site following low back, neck, shoulder, or knee surgery. Study participants had received various treatments for pain before and after surgery, and their level of satisfaction regarding surgery was low. Meanwhile, their preference for KM interventions was relatively high. Particularly, low back surgery patients reported high ODI scores, which indicates that prognosis after low back surgery tends to be poor.

As no age limit was set when recruiting patients, those in any age group were eligible for participation. However, an analysis of sociodemographic characteristics showed that the highest number of patients belonged to the 60–69 and 50–59 years' groups in both the low back and other surgery groups. This finding can be interpreted from two perspectives. First, it may be taken to indicate that many elderly patients in Korea experience chronic postsurgical pain. That a high percentage of participants had hypertension and that almost 50% were unemployed are also indicative of the fact that a high proportion of participants were elderly individuals. Second, it could be taken to indicate that elderly patients aged over 50 years prefer KM hospitals, which is in line with previous reports [25].

In general, surgery is more expensive than nonsurgical interventions [11], although it may be more cost-effective at certain timepoints [26, 27]. A major point that the authors wish to draw attention to in the present study is that patients who received orthopedic surgery had to pay not only for surgery, but also for substantial medical costs incurred before and after surgery. In the present study, the items that accounted for the highest proportion of postsurgical medical costs were associated with minimally invasive surgical procedures (5,783.3 thousand Korean Won/person) and resurgery (4,543.2 thousand Korean Won/person). Meanwhile, most patients received MRI and X-ray examinations after surgery, and the most common postsurgical interventions were medication and physical therapy. MRIs are generally used as diagnostic tools to identify red flags such as spondylitis or metastasis and perform differential diagnosis in patients with low back pain [28], but they are also used to determine cause of recurrence and to check prognosis. In the present study, 73.2% of low back surgery patients (*n* = 82) responded that they underwent MRI examinations after surgery. Moreover, the results show that incurrence of substantial medical costs continued for postsurgical care, as indicated by 57% of participants spending approximately 547.8 thousand Korean Won per person on medication and 46% spending 422.0 thousand Korean Won per person on physical therapy.

Minimally invasive procedures, including percutaneous epidural neuroplasty [29], intradiscal electrothermal therapy, and discoplasty [30], are not covered by national health insurance in Korea. These minimally invasive procedures are usually considered by patients who wish to choose an alternative to open surgery or in cases where open surgical treatment may not be feasible. However, the present findings suggest that such procedures can be associated with greater economic burden. Meanwhile, most spinal injections, such as facet joint blocks, epidural nerve blocks, and selective nerve blocks, are covered by national health insurance. Accordingly, the deductibles for such injection therapies (total cost: 240.0 thousand Korean Won) would be much lower than those for other surgical interventional therapies.

Chronic pain after low back surgery is being discussed with consideration for failed back surgery syndrome (FBSS). FBSS refers to low back or radiating leg pain that recurs or persists after surgery [5]. FBSS treatments may vary depending on the cause, and resurgery may be considered as an option [31]. In the present study, 25.6% of patients who received low back surgery underwent resurgery, with an average of 1.3 surgeries, which is similar to results from a previous study reporting that approximately 20–40% of cases are subject to resurgery due to FBSS [32]. Among participants in the present study, those who underwent resurgery for low back pain paid high costs of 4,732.5 (4,162.2) thousand Korean Won for resurgery. Moreover, patients with FBSS have a lower success rate with resurgery [15] and may experience postsurgical complications, such as bleeding and infections [33]. Together, these findings indicate that resurgery due to FBSS or CPCS may unnecessarily expose patients to additional risk and burden.

Most KM interventions using acupuncture, cupping, and moxibustion had relatively low cost per treatment, which is possible as these treatments are covered by Korean national health insurance [34]. On the other hand, pharmacopuncture, Chuna manual medicine, and herbal decoctions are not covered by national health insurance, and, as a result, these treatments were more expensive, and fewer patients chose such interventions. While patient preference and expectancy need to be duly considered in interpreting these results, they are indicative of satisfaction with CAM medicine treatment and outcomes as participants showed a high level of satisfaction with KM and used KM interventions before and after surgery.

With respect to the health status of participants on the day of the survey based on self-report, the participants showed a mean NRS score of 6.0 ± 2.2 points for pain, EQ-5D-5L score of 0.6 ± 0.2 for quality of life, BDI-II score of 15.3 ± 10.2 points for depression, ODI score of 40.1 ± 19.2 points for low back dysfunction, and daily activity impairment score of 5.9 ± 2.6%. According to an existing study, the estimated ODI cut-off value for differentiating between patients with low back pain and healthy people was 12 points, and the mean ODI score for low back pain patients was 22.1 points [35]. These results suggest that the patients with postsurgical low back pain included in the present study had more severe dysfunction compared to typical low back pain patients, suggesting that their high mean BDI-II scores should be examined further. The BDI-II, an updated version of the original BDI which was revised in 1996, is useful for the initial assessment of presence of depressive symptoms and follow-up of patients suspected of having depression [23]. It is well known that persistent pain can lead to psychiatric problems through loss of sleep and mood changes. Therefore, the high BDI-II scores observed in the participants with chronic postsurgical pain are not surprising. However, in contrast to high mean values, only four participants had been diagnosed with depression or were currently being treated for depression, which suggests the need for a multidisciplinary approach not only to relieve pain, but also to treat psychiatric symptoms, such as depression, in patients after surgery.

The participants in the present study showed a mean satisfaction score for surgery of 5.5 ± 2.8 points on a 9-point Likert scale, which was in contrast to many reports of patient satisfaction with surgery in surveys conducted immediately after surgery. For example, in a study conducted on patients who underwent surgery under general or local anesthesia at a university hospital, 95.2% of 353 respondents reported that they were satisfied with the anesthesia administration and surgery [36]. However, the same study also revealed that, among the major causes of patient dissatisfaction after surgery, postsurgical pain accounted for the largest portion. Meanwhile, with respect to long-term surgery satisfaction after spinal surgery, studies of 8–10 years' follow-up period in patients with disc disorder or stenosis [37, 38] reported that patients with stenosis who received surgical treatment and those who received nonsurgical treatment showed comparable levels of medical service satisfaction, whereas those with disc disorders who received surgical treatment were more satisfied with their current status. Among patients with knee disorders, those who received meniscectomy reported favorable prognosis in an 8-year follow-up [39].

Meanwhile, studies on the efficacy of CAM for postsurgical pain have mostly been limited to use of acupuncture. A previous study on 1,700 patients who underwent hip or knee replacement surgery reported that acupuncture therapy was effective in reducing pain [40], while another study reported that combining acupuncture with existing therapeutic modalities during arthroscopic shoulder surgery was effective in reducing pain [41]. On the other hand, a 2014 systematic literature review reported that the effects of acupuncture after surgery were controversial [42], while the postsurgical pain treatment guidelines by the American Pain Society neither recommended nor discouraged acupuncture and massage [43].

The present study holds several strengths. First, data was collected from postsurgical pain patients from four centers located in four major cities in Korea. The participants were included evenly nationwide across all study locations, and the study prescreened over 16,000 patients as eligible participants. The present study also extensively and rigorously collected data on current health status and medical expenditure including out-of-pocket expenses for noninsurance items. Moreover, the study also obtained medical service satisfaction and health-related information through direct patient responses, which can only be roughly estimated in use of administrative data. Thus, more detailed and comprehensive information about patient health status was obtained.

However, the present study also holds various limitations. First, as a survey study, it did not use medical records or insurance or administrative data, and instead it used self-reported outcomes. As such, it is susceptible to recall bias and it was not possible to analyze patient condition based on objective findings, such as test results. Second, several patients had a long-term prognosis after surgery, and there were cases in which it was unclear which treatments were received, whether received treatments were postsurgically relevant or for separate pain episodes unrelated to surgery, or which classification a specific treatment item was categorized under. To minimize confusion between the participants and researchers, researchers followed a preestablished standard operating procedure. Third, because only patients who voluntarily visited KM hospitals were considered as eligible participants, responses on utilization rate and level of satisfaction may be biased in favor of KM due to selection bias, and this aspect needs to be given due consideration in interpretation of results. Fourth, in analysis of medical costs, if the time of actual expenditure was known, annual average inflation rates could have been applied for standardization of cost, but this was precluded in the current study due to its inherent limitation as a cross-sectional study compiling collective data retrospectively over a certain period of time.

## 5. Conclusions

In conclusion, these findings show tendencies for low satisfaction regarding surgery and high preference for KM-based interventions. It can be inferred that it is therefore important to provide appropriate pre- and postsurgical care for patients with musculoskeletal pain to improve pain, function, and quality of life. Furthermore, using KM interventions in conjunction with conventional interventions after surgery may be an effective option to consider for relieving pain and improving function and quality of life in this patient population.

## Figures and Tables

**Table 1:** Sociodemographic characteristics.

Factor		Total (*n* = 100)	Back pain (*n* = 82)	Other sites of pain (*n* = 18)
Age (years)	Mean (SD)	52.58 (15.4)	53.20 (15.8)	49.78 (13.6)
	20–30, *n* (%)	10 (10.0)	8 (9.8)	2 (11.1)
	30–40, *n* (%)	14 (14.0)	12 (14.6)	2 (11.2)
	40–50, *n* (%)	20 (20.0)	17 (20.7)	3 (16.7)
	50–60, *n* (%)	14 (14.0)	7 (8.5)	7 (38.9)
	>60, *n* (%)	42 (42.0)	38 (46.3)	4 (22.2)

Sex, *n* (%)	Male	52 (52.0)	43 (52.4)	9 (50.0)
	Female	48 (48.0)	39 (47.6)	9 (50.0)

Comorbidities, *n* (%)	Hypertension	31 (31.0)	28 (34.1)	3 (16.7)
	Diabetes	11 (11.0)	11 (13.4)	0 (0.0)
	Depression	4 (4.0)	3 (3.7)	1 (5.6)
	Cardiovascular	11 (11.0)	9 (11.0)	2 (11.1)
	Pulmonary	3 (3.0)	3 (3.7)	0 (0.0)
	Gastrointestinal	5 (5.0)	2 (2.4)	3 (16.7)

Employment, *n* (%)	Employed	49 (49.0)	37 (45.1)	12 (66.7)
	Unemployed	51 (51.0)	45 (54.9)	6 (33.3)

Alcohol consumption, *n* (%)	Yes	50 (50.0)	39 (47.6)	11 (61.1)
	No	50 (50.0)	43 (52.4)	7 (38.9)

Average alcohol consumption units per week (*n* = 50)	Mean (SD)	8.16 (7.5)	8.18 (7.7)	8.09 (7.4)

Smoking status, *n* (%)	Current smoker	24 (24.2)	17 (21.0)	7 (38.9)
	Nonsmoker	75 (75.8)	64 (79.0)	11 (61.1)

Average cigarettes smoked per day (*n* = 24)	Mean (SD)	17.58 (8.4)	17.94 (7.3)	16.71 (11.4)

Cause of initial pain episode onset	Daily activity	59 (59.0)	49 (59.8)	10 (55.6)
	Work or exercise	31 (31.0)	26 (31.7)	5 (27.8)
	Accident (traffic, etc.)	10 (10.0)	7 (8.5)	3 (16.7)

Average cost of initial surgery (Korean Won/1,000)	Mean (SD)	4,112.5 (2,655.8)	4,217.3 (2,770.4)	3,635.0 (2,052.2)
Average total costs (Korean Won/1,000)	Mean (SD)	6846.0 (10,442.6)	7,525.3 (11,333.2)	3,751.7 (3,270.6)

SD, standard deviation; 1,200 Korean Won = 1 US Dollar.

**Table 2:** Utilization of medical resources before surgery.

		Total (*n* = 100)	Back pain (*n* = 82)	Other sites of pain (*n* = 18)
*Diagnostic imaging*				
Radiology	No. of patients, *n* (%)	60 (60.0)	46 (56.1)	14 (77.8)
	No. of treatments, mean (SD)	2.4 (2.8)	2.3 (2.9)	2.4 (2.4)
	Treatment cost (Korean Won/1,000), mean (SD)	28.0 (32.7)	28.1 (35.3)	27.6 (23.8)
Computed tomography	No. of patients, *n* (%)	36 (36.0)	29 (35.4)	7 (38.9)
	No. of treatments, mean (SD)	1.1 (0.7)	1.1 (0.7)	1.1 (0.4)
	Treatment cost (Korean Won/1,000), mean (SD)	153.8 (112.4)	151.4 (114.7)	162.9 (111.8)
Magnetic resonance imaging	No. of patients, *n* (%)	89 (89.0)	73 (89.0)	16 (88.9)
	No. of treatments, mean (SD)	1.3 (0.7)	1.3 (0.7)	1.3 (0.7)
	Treatment cost (Korean Won/1,000), mean (SD)	547.9 (317.9)	542.1 (305.0)	573.8 (380.8)
Ultrasound imaging	No. of patients, *n* (%)	4 (4.0)	1 (1.2)	3 (16.7)
	No. of treatments, mean (SD)	1.0 (0.0)	1.0 (-)	1.0 (0.0)
	Treatment cost (Korean Won/1,000), mean (SD)	55.0 (30.0)	90.0 (-)	43.3 (23.1)

*Conventional interventions*				
Medications	No. of patients, *n* (%)	34 (34.0)	23 (28.0)	11 (61.1)
	Treatment duration (day), mean (SD)	114.9 (309.4)	65.6 (95.3)	217.9 (527.8)
	Treatment cost (Korean Won/1,000), mean (SD)	293.3 (491.1)	267.9 (443.0)	343.9 (596.0)
Physical therapies	No. of patients, *n* (%)	43 (43.0)	33 (40.2)	10 (55.6)
	No. of treatments, mean (SD)	63.2 (248.4)	71.2 (282.6)	36.9 (68.8)
	Treatment cost (Korean Won/1,000), mean (SD)	662.1 (2,469.5)	809.3 (2,820.2)	191.3 (250.6)
Manual therapies	No. of patients, *n* (%)	14 (14.0)	10 (12.2)	4 (22.2)
	No. of treatments, mean (SD)	72.3 (221.9)	82.8 (251.2)	37.6 (60.3)
	Treatment cost (Korean Won/1,000), mean (SD)	1,075.0 (1,009.9)	1,166.0 (1,093.7)	847.5 (857.0)
Injections covered under insurance	No. of patients, *n* (%)	28 (28.0)	21 (25.6)	7 (38.9)
	No. of treatments, mean (SD)	4.8 (6.8)	5.1 (7.7)	4.1 (2.9)
	Treatment cost (Korean Won/1,000), mean (SD)	212.5 (222.5)	215.7 (170.8)	202.9 (354.2)
Minimally invasive surgical treatments	No. of patients, *n* (%)	11 (11.0)	9 (11.0)	2 (11.1)
	No. of treatments, mean (SD)	1.36 (0.9)	1.44 (1.0)	1.0 (0.0)
	Treatment cost (Korean Won/1,000), mean (SD)	3,104.6 (2,090.7)	3,500.0 (2107.1)	1,325.0 (671.8)

*Korean medicine interventions*				
Acupuncture	No. of patients, *n* (%)	48 (48.0)	39 (47.6)	9 (50.0)
	No. of treatments, mean (SD)	51.3 (81.1)	56.8 (88.1)	27.7 (31.9)
	Treatment cost (Korean Won/1,000), mean (SD)	445.6 (1,339.2)	510.6 (1,478.8)	164.1 (197.1)
Pharmacopuncture	No. of patients, *n* (%)	17 (17.0)	13 (15.9)	4 (22.2)
	No. of treatments, mean (SD)	36.2 (61.6)	43.2 (69.0)	13.3 (17.9)
	Treatment cost (Korean Won/1,000), mean (SD)	930.0 (1,722.5)	1,113.8 (1,927.8)	332.5 (578.4)
Herbal decoctions	No. of patients, *n* (%)	14 (14.0)	12 (14.6)	2 (11.1)
	Treatment duration (day), mean (SD)	98.9 (106.7)	110.8 (111.2)	27.5 (17.7)
	Treatment cost (Korean Won/1,000), mean (SD)	3,229.4 (4,248.0)	3676.0 (4,448.9)	550.0 (353.6)
Cupping	No. of patients, *n* (%)	35 (35.0)	27 (32.9)	8 (44.4)
	No. of treatments, mean (SD)	54.2 (75.7)	62.4 (82.8)	26.8 (34.9)
	Treatment cost (Korean Won/1,000), mean (SD)	464.1 (1,543.6)	566.3 (1,749.8)	119.2 (158.0)
Moxibustion	No. of patients, *n* (%)	8 (8.0)	7 (8.5)	1 (5.6)
	No. of treatments, mean (SD)	40.5 (45.5)	43.4 (48.3)	20.0 (-)
	Treatment cost (Korean Won/1,000), mean (SD)	226.7 (518.5)	256.2 (552.7)	20.0 (-)

Chuna manual therapies	No. of patients, *n* (%)	11 (11.0)	10 (12.2)	1 (5.6)
	No. of treatments, mean (SD)	44.5 (71.8)	44.9 (75.7)	40.0 (-)
	Treatment cost (Korean Won/1,000), mean (SD)	1,303.6 (2,301.6)	1,314.0 (2,425.8)	1,200.0 (-)

SD, standard deviation; 1,200 Korean Won = 1 US Dollar.

**Table 3:** Utilization of medical resources after surgery.

		Total (*n* = 100)	Back pain (*n* = 82)	Other sites of pain (*n* = 18)
*Diagnostic imaging*				
Radiology	No. of patients, *n* (%)	52 (52.0)	43 (52.4)	9 (50.0)
	No. of treatments, mean (SD)	3.2 (3.0)	3.0 (2.9)	4.4 (3.5)
	Treatment cost (Korean Won/1,000), mean (SD)	39.2 (38.1)	37.9 (40.5)	45.3 (25.1)
Computed tomography	No. of patients, *n* (%)	14 (14.0)	11 (13.4)	3 (16.7)
	No. of treatments, mean (SD)	1.2 (0.6)	1.27 (0.6)	1.0 (0.0)
	Treatment cost (Korean Won/1,000), mean (SD)	186.4 (76.4)	196.4 (81.2)	150.0 (50.0)
Magnetic resonance imaging	No. of patients, *n* (%)	68 (68.0)	60 (73.2)	8 (44.4)
	No. of treatments, mean (SD)	1.6 (1.1)	1.7 (1.1)	1.5 (0.8)
	Treatment cost (Korean Won/1,000), mean (SD)	725.7 (542.7)	721.9 (563.6)	753.8 (379.1)
Ultrasound imaging	No. of patients, *n* (%)	3 (3.0)	2 (2.4)	1 (5.6)
	No. of treatments, mean (SD)	1.0 (0.0)	1.0 (0.0)	1.00 (-)
	Treatment cost (Korean Won/1,000), mean (SD)	46.7 (20.8)	55.0 (21.2)	30.00 (-)

*Conventional interventions*				
Medications	No. of patients, *n* (%)	57 (57.0)	44 (54.3)	13 (72.2)
	Treatment duration (day), mean (SD)	126.4 (351.7)	141.5 (396.3)	75.4 (104.4)
	Treatment cost (Korean Won/1,000), mean (SD)	547.8 (1810.8)	668.5 (2048.8)	139.3 (161.5)
Physical therapies	No. of patients, *n* (%)	46 (46.0)	38 (46.3)	8 (44.4)
	No. of treatments, mean (SD)	58.7 (104.6)	63.0 (113.2)	38.6 (45.2)
	Treatment cost (Korean Won/1,000), mean (SD)	422.0 (1,033.6)	431.6 (1,096.5)	377.5 (727.2)
Manual therapies	No. of patients, *n* (%)	25 (25.0)	18 (22.0)	7 (38.9)
	No. of treatments, mean (SD)	21.7 (39.7)	24.3 (46.4)	14.9 (11.2)
	Treatment cost (Korean Won/1,000), mean (SD)	1,784.6 (4,040.2)	2,219.7 (4,716.1)	665.7 (495.5)
Injections covered under insurance	No. of patients, *n* (%)	39 (39.0)	31 (37.8)	8 (44.4)
	No. of treatments, mean (SD)	5.3 (9.7)	3.2 (2.9)	13.5 (19.4)
	Treatment cost (Korean Won/1,000), mean (SD)	240.0 (286.3)	217.6 (306.2)	326.9 (179.9)
Minimally invasive surgical treatments	No. of patients, *n* (%)	6 (6.0)	3 (3.7)	3 (16.7)
	No. of treatments, mean (SD)	2.8 (1.5)	2.67 (1.2)	3.0 (2.0)
	Treatment cost (Korean Won/1,000), mean (SD)	5,783.3 (2,958.7)	7,666.7 (2,081.7)	3,900.0 (2,628.7)
Resurgeries	No. of patients, *n* (%)	23 (23.0)	21 (25.6)	2 (11.1)
	No. of treatments, mean (SD)	1.3 (0.6)	1.3 (0.6)	1.0 (0.0)
	Treatment cost (Korean Won/1,000), mean (SD)	4,543.2 (4,021.9)	4,732.5 (4,162.2)	2,650.0 (1,626.3)

*Korean medicine interventions*				
Acupuncture	No. of patients, *n* (%)	52 (52.0)	46 (56.1)	6 (33.3)
	No. of treatments, mean (SD)	114.2 (422.3)	127.4 (447.9)	13.5 (8.6)
	Treatment cost (Korean Won/1,000), mean (SD)	627.0 (2,576.4)	698.3 (2,734.5)	36.5 (79.8)
Pharmacopuncture	No. of patients, *n* (%)	32 (32.0)	27 (32.9)	5 (27.8)
	No. of treatments, mean (SD)	35.8 (60.4)	41.2 (64.5)	6.8 (3.1)
	Treatment cost (Korean Won/1,000), mean (SD)	776.4 (1,673.3)	907.5 (1,795.5)	68.0 (31.1)
Herbal decoctions	No. of patients, *n* (%)	18 (18.0)	17 (20.7)	1 (5.6)
	No. of treatments, mean (SD)	88.2 (88.8)	92.5 (89.6)	15.0 (-)
	Treatment cost (Korean Won/1,000), mean (SD)	1,880.0 (2,180.1)	1,973.0 (2,210.1)	300.0 (-)
Cupping	No. of patients, *n* (%)	38 (38.0)	33 (40.2)	5 (27.8)
	No. of treatments, mean (SD)	136.7 (492.7)	156.0 (527.1)	9.2 (1.8)
	Treatment cost (Korean Won/1,000), mean (SD)	535.6 (2,140.4)	610.3 (2,292.0)	42.8 (14.8)
Moxibustion	No. of patients, *n* (%)	8 (8.0)	7 (8.5)	1 (5.6)
	No. of treatments, mean (SD)	57.1 (69.1)	63.9 (71.7)	10.0 (-)
	Treatment cost (Korean Won/1,000), mean (SD)	137.7 (175.1)	156.0 (180.8)	10.0 (-)
Chuna manual therapies	No. of patients, *n* (%)	14 (14.0)	13 (15.9)	1 (5.6)
	No. of treatments, mean (SD)	38.1 (53.2)	40.9 (54.4)	3.0 (-)
	Treatment cost (Korean Won/1,000), mean (SD)	1,218.9 (1,668.9)	1,305.8 (1,703.8)	90.0 (-)

SD, standard deviation; 1,200 Korean Won = 1 US Dollar.

**Table 4:** Medical service satisfaction and health-related scales.

		Total (*n* = 100)	Back pain (*n* = 82)	Other sites of pain (*n* = 18)
Medical service satisfaction, mean (SD)	Initial surgery	5.5 (2.8)	5.6 (2.8)	5.0 (2.5)
	Diagnostic imaging	6.4 (2.4)	6.2 (2.5)	6.9 (1.9)
	Conventional interventions	5.2 (2.0)	5.1 (2.1)	5.4 (1.5)
	Korean medicine tests	5.5 (1.8)	5.4 (1.8)	5.9 (1.6)
	Korean medicine interventions	6.3 (1.7)	6.2 (1.8)	6.8 (1.2)

Health-related scales, mean (SD)	NRS	6.0 (2.2)	6.1 (2.2)	5.3 (2.3)
	EQ-5D-5L	0.6 (0.2)	0. 6 (0.2)	0.7 (0.2)
	EQ-VAS	53.0 (20.3)	51.1 (19.3)	61.9 (22.6)
	BDI-II	15.3 (10.2)	15.1 (10.5)	16.2 (9.3)
	Functional disability scales	—	ODI: 40.1 (19.2)	NDI (*n* = 7): 33.1 (19.9);
				WOMAC (*n* = 10): 37.8 (20.3);
				SPADI (*n* = 1): 81.500 (-)
	WPAI-GH: overall work productivity impairment due to health (%)	62.5 (47.8)	65.3 (47.0)	49.7 (50.7)
	WPAI-GH: overall activity impairment due to health (%)	5.9 (2.6)	6.1 (2.5)	5.2 (2.8)

SD, standard deviation; NRS, Numeric Rating Scale, EQ-5D-5L, 5-Level EuroQol-5 Dimension; EQ-VAS, EuroQol-Visual Analogue Scale; BDI-II, Beck's Depression Index II; ODI, Oswestry Disability Index; NDI, Neck Disability Index; WOMAC, Western Ontario and McMaster Universities Arthritis Index; SPADI, Shoulder Pain and Disability Index; WPAI-GH, Work Productivity and Activity Impairment Questionnaire: General Health.

## Data Availability

Data used to support the findings of this study are available from the corresponding author upon request.

## Abbreviations

BDI-II: Beck's Depression Index II
CAM: Complementary and alternative medicine
CPSP: Chronic postsurgical pain
EQ-5D-5L: 5-Level EuroQol-5 Dimension
EQ-VAS: EuroQol-Visual Analogue Scale
FBSS: Failed back surgery syndrome
IRB: Institutional Review Board
JHKM: Jaseng Hospital of Korean Medicine
KM: Korean medicine
NDI: Neck Disability Index
NRS: Numeric Rating Scale
NSAIDs: Nonsteroidal anti-inflammatory drugs
ODI: Oswestry Disability Index
SPADI: Shoulder Pain and Disability Index
WOMAC: Western Ontario and McMaster Universities Arthritis Index
WPAI-GH: Work Productivity and Activity Impairment Questionnaire: General Health.
